# Genomic Islands in the Pathogenic Filamentous Fungus *Aspergillus fumigatus*

**DOI:** 10.1371/journal.pgen.1000046

**Published:** 2008-04-11

**Authors:** Natalie D. Fedorova, Nora Khaldi, Vinita S. Joardar, Rama Maiti, Paolo Amedeo, Michael J. Anderson, Jonathan Crabtree, Joana C. Silva, Jonathan H. Badger, Ahmed Albarraq, Sam Angiuoli, Howard Bussey, Paul Bowyer, Peter J. Cotty, Paul S. Dyer, Amy Egan, Kevin Galens, Claire M. Fraser-Liggett, Brian J. Haas, Jason M. Inman, Richard Kent, Sebastien Lemieux, Iran Malavazi, Joshua Orvis, Terry Roemer, Catherine M. Ronning, Jaideep P. Sundaram, Granger Sutton, Geoff Turner, J. Craig Venter, Owen R. White, Brett R. Whitty, Phil Youngman, Kenneth H. Wolfe, Gustavo H. Goldman, Jennifer R. Wortman, Bo Jiang, David W. Denning, William C. Nierman

**Affiliations:** 1 The J. Craig Venter Institute, Rockville, Maryland, United States of America; 2 Department of Genetics, Smurfit Institute, University of Dublin, Trinity College, Dublin, Ireland; 3 School of Medicine and Faculty of Life Sciences, University of Manchester, Manchester, United Kingdom; 4 Department of Biology, McGill University, Montreal, Quebec, Canada; 5 Agricultural Research Service, United States Department of Agriculture, Department of Plant Sciences, University of Arizona, Tucson, Arizona, United States of America; 6 School of Biology, University of Nottingham, Nottingham, United Kingdom; 7 Institute for Research in Immunology and Cancer, Department of Computer Science and Operations Research, Universite de Montreal, Montreal, Canada; 8 Departamento de Ciencias Farmaceuticas, Faculdade de Ciencias Farmaceuticas de Ribeirao Preto, Universidade de Sao Paulo, Sao Paulo, Brazil; 9 Merck & Co., Inc., Whitehouse Station, New Jersey, United States of America; 10 Department of Molecular Biology and Biotechnology, The University of Sheffield, Sheffield, United Kingdom; 11 Department of Biochemistry and Molecular Biology, The George Washington University School of Medicine, Washington DC, United States of America; Department of Energy Joint Genome Institute, United States of America

## Abstract

We present the genome sequences of a new clinical isolate of the important human pathogen, *Aspergillus fumigatus*, A1163, and two closely related but rarely pathogenic species, *Neosartorya fischeri* NRRL181 and *Aspergillus clavatus* NRRL1. Comparative genomic analysis of A1163 with the recently sequenced *A. fumigatus* isolate Af293 has identified core, variable and up to 2% unique genes in each genome. While the core genes are 99.8% identical at the nucleotide level, identity for variable genes can be as low 40%. The most divergent loci appear to contain heterokaryon incompatibility (*het*) genes associated with fungal programmed cell death such as developmental regulator *rosA*. Cross-species comparison has revealed that 8.5%, 13.5% and 12.6%, respectively, of *A. fumigatus*, *N. fischeri* and *A. clavatus* genes are species-specific. These genes are significantly smaller in size than core genes, contain fewer exons and exhibit a subtelomeric bias. Most of them cluster together in 13 chromosomal islands, which are enriched for pseudogenes, transposons and other repetitive elements. At least 20% of *A. fumigatus*-specific genes appear to be functional and involved in carbohydrate and chitin catabolism, transport, detoxification, secondary metabolism and other functions that may facilitate the adaptation to heterogeneous environments such as soil or a mammalian host. Contrary to what was suggested previously, their origin cannot be attributed to horizontal gene transfer (HGT), but instead is likely to involve duplication, diversification and differential gene loss (DDL). The role of duplication in the origin of lineage-specific genes is further underlined by the discovery of genomic islands that seem to function as designated “gene dumps” and, perhaps, simultaneously, as “gene factories”.

## Author Summary

*Aspergillus* is an extremely diverse genus of filamentous ascomycetous fungi (molds) found ubiquitously in soil and decomposing vegetation. Being supreme opportunists, aspergilli have adapted to overcome various chemical, physical, and biological stresses found in heterogeneous environments. While most species in the genus are saprophytes, a surprising number are able to infect wounded plants and animals. Remarkably, the allergic human host also responds abnormally to the aspergilli with lung and sinus disease. The advent of immunosuppressive agents and other medical advances have created a large worldwide pool of human hosts susceptible to some *Aspergillus* species, including the world's most harmful mold and the causative agent of invasive aspergillosis, *Aspergillus fumigatus*. In this study, we have used the power of comparative genomics to gain insight into genetic mechanisms that may contribute to the metabolic versatility and pathogenicity of this important human pathogen. Comparison of the genomes of two *A. fumigatus* clinical isolates and two closely related, but rarely pathogenic species showed that their genomes contain several large isolate- and species-specific chromosomal islands. The metabolic capabilities encoded by these highly labile regions are likely to contribute to their rapid adaptation to heterogeneous environments such as soil or a living host.

## Introduction

*Aspergillus fumigatus* is exceptional amongst the aspergilli in being both a primary and opportunistic pathogen as well as a major allergen associated with severe asthma and sinusitis [1]–[3]. It was first reported to cause opportunistic invasive infection about 50 years ago [4]. In immunocompromised patients, mycelial growth can proliferate throughout pulmonary or other tissues causing invasive aspergillosis. For these patients, the incidence of invasive aspergillosis can be as high as 50% and the mortality rate is often 50%, even with antifungal treatment. Since the late 1800's [2], *A. fumigatus* has been demonstrated to be a primary pathogen of the airways, sinuses, lungs, damaged skin and subcutaneous tissues. For example, it can cause post-operative infection in all human organs [5]. In most cases diagnosis remains problematic and can compromise effective medical treatment.

*A. fumigatus* is thought to possess particular metabolic capabilities and genetic determinants that allow it to initiate and establish an *in vivo* infection. This conclusion is supported by the observation that the majority of invasive aspergillosis disease is caused by *A. fumigatus*, even though its conidia comprise only a small percentage of the total conidia found in air-sampling studies [6]. While the interaction of *A. fumigatus* spores with the human respiratory mucosa is understood to an extent, the basic biology of the organism has until recently received little attention.

**Figure 1:**
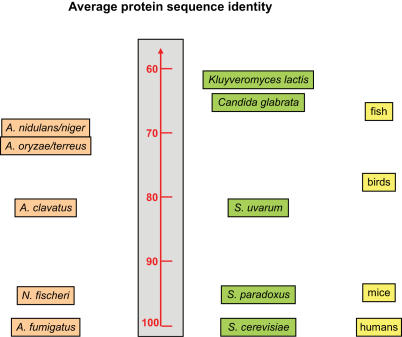
Molecular Divergence in Molds and Yeasts. A. fumigatus proteins are compared to their orthologs in *N. fischeri*, *A. clavatus*, *A. terreus*, *A. oryzae*, *A. nidulans*, and *A. niger* (mean values: 95%, 84%, 71%, 71%, 68%, and 69%, respectively). *Saccharomyces paradoxus*, *Saccharomyces uvarum*, *Candida glabrata*, and *Kluyveromyces lactis* are compared to *Saccharomyces cerevisiae* (adapted from [74,75]). Mean values for these species are 90%, 82%, 64%, and 60%, respectively. Median percent identity between pairs of orthologs from *A. fumigatus* and each successive genome in the tree is shown. Relative divergence of humans, mice, birds and fish are shown for reference.

**Figure 2:**
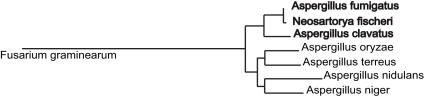
Three Closely Related Aspergilli. The three most closely related aspergilli, which constitute the Affc-core group (*A. fumigatus*, *N. fischeri*, and *A. clavatus*), are in bold black. The maximum-likelihood tree was constructed from an alignment of 90 proteins chosen on the basis of similar lengths and identical number of intron/exon structures in order to minimize the number of inconvenient or incongruent gene models (see Materials and Methods).

Recently we presented the genomic sequence of *A. fumigatus* strain Af293 (FGSC A1100) [7] isolated from a neutropenic patient, who died from invasive aspergillosis [8]. Its comparison with the genomes of two distantly related species, *Aspergillus nidulans* and *Aspergillus oryzae*, has led to many unexpected discoveries, including the possibility of a hidden sexual cycle in *A. fumigatus* and *A. oryzae*, and the detection of remarkable genetic variability of this genus [9],[10]. Although members of the same genus, these three species are approximately as evolutionarily distant from each other at the molecular level as humans and fish (Figures 1 and 2) [11]. This significant phylogenetic distance has hindered some aspects of comparative genomic analysis of the aspergilli such as identification of the genetic traits responsible for differences in virulence as well as in sexual and physiological properties.

To maximize the resolving power of whole-genome comparative analysis, we selected the environmental type strains of a very closely related sexual species, *Neosartorya fischeri* NRRL181 (*A. fischerianus*), and a more distantly related asexual species, *A. clavatus* NRRL1, for complete sequencing. These three species are referred to here as the Affc lineage for *A. fumigatus*, *N. fischeri*, and *A. clavatus* (Figure 2). In contrast to *A. fumigatus*, *N. fischeri* is only rarely identified as a human pathogen [12]–[15]; while *A. clavatus* is probably an important allergen and the causative agent of extrinsic allergic alveolitis known as malt worker's lung [16]. *A. clavatus* also produces a number of mycotoxins and has been associated with neurotoxicosis in sheep and cattle fed infected grain worldwide (e.g. [17]). Our phenotypic characterization (Table S1) has shown that both *A. fumigatus* and *N. fischeri* can grow at 42°C, which indicates that *A. fumigatus* may possess other genetic determinants besides thermotolerance that allow it to establish a successful *in vivo* infection.

As determined by multilocus sequence comparison, most *A. fumigatus* isolates, including Af293 and A1163, lie within the main *A. fumigatus* clade and persist as a single, global phylogenetic population, presumably due to its small spore size [18]. Natural *A. fumigatus* isolates were described previously as having low genetic diversity in comparison to *N. fischeri* isolates [19]. However recent studies identified a number of strain-specific [7] and polymorphic [20],[21] genes. To further explore the extent of genetic variation within the *A. fumigatus* species, we included in this analysis the genome sequence of a second strain, A1163, made available through Merck & Co., Inc., Whitehouse Station, NJ. Our preliminary analysis has shown that Af293 and A1163 isolates vary greatly in their resistance to antifungals (Table S2).

## Results/Discussion

### *A. fumigatus* Af293 vs. *A. fumigatus* A1163

**Figure 3:**
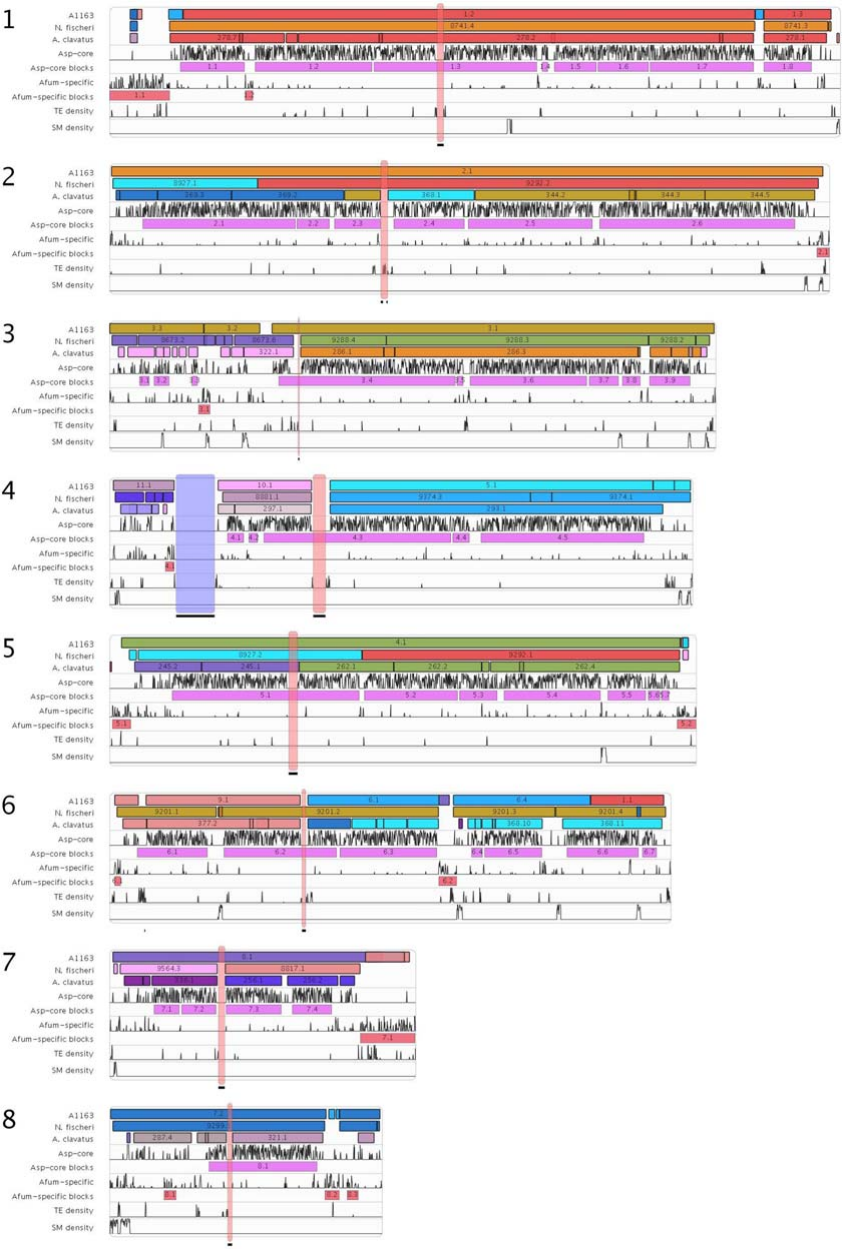
Alignment of the A1163, *N. fischeri*, and *A. clavatus* Assemblies against the Eight Af293 Chromosomes. The first three tracks from the top for each reference chromosome show syntenic blocks (horizontal bars) identified in the target genomes, *A. fumigatus* A1163, *N. fischeri*, and *A. clavatus*. Each assembly from the target genomes is represented by a single color. Syntenic blocks are numbered based on the target genome assembly ID and the position of the block in the target genome assembly. Tracks 4 and 5 show Asp-core gene density and blocks (horizontal bars), respectively, in the Af293 genome. Tracks 6 and 7 show Afum-specific gene density and blocks (horizontal bars), respectively. Tracks 8 and 9 show the density of clustered secondary metabolite biosynthesis genes and transposable elements, respectively, found in Af293. Pink vertical bars represent putative centromeres, the purple vertical bar in chromosome 4 represents a region of ribosomal DNA, and horizontal black bars beneath each chromosome designate sequencing gaps.

The genome of *A. fumigatus* strain A1163 was sequenced by the whole genome random sequencing method [22]. Its genome (29.2 Mb) is 1.4% larger than the genome of the first sequenced strain Af293 (28.8 Mb) (Table 1). About 98% of each genome can be aligned with high confidence. Alignment of the A1163 genome against the eight Af293 chromosomes has revealed 17 large syntenic blocks, which correspond roughly to the 16 Af293 chromosomal arms (Figure 3). The syntenic blocks were defined as regions containing at least five syntenic orthologs separated by no more than 20 genes without orthologs.

**Table 1:** Genome Statistics

Sequenced organisms	Af293	A1163	*N. fischeri*	*A. clavatus*
Length (Mp)	28.810	29.205	32.552	27.859
Assemblies >100 Kb	18	11	13	16
GC content	50%	49%	49%	49%
No. of genes	9631	9906	10407	9125
No. of LS genes	818		1408	1151
Mean gene length (Bp)	1478	1455	1466	1483
% Genes with introns	79%	80%	80%	81%
% Coding	49%	49%	47%	49%

Most translocation events involving *A. fumigatus* chromosomes appear to have taken place within 300 Kb from the telomeres. The largest exchange involved a ∼500 Kb segment between Af293 chromosomes 1 and 6 and A1163, which contain regions aligning with A1163 assembly 1 (syntenic blocks 1.1 and 1.2 in Figure 3). This appears to be a recent event that happened in A293. In addition, Af293 chromosome 1 harbours a 400 Kb subtelomeric region that does not align well with A1163 assemblies. There is evidence of gene conversion between distal subtelomeric sequences encoding RecQ family helicases in *A. fumigatus* chromosomes 2, 4, and 7.

Consistent with previous reports [19], the identity over the shared regions is very high (99.8% at the nucleotide level). This is higher than 99.3% and 99.5% identity between the two sequenced *A. niger* isolates (ATCC 1015 and CBS 513.88) [23] and between *A. oryzae* [10] and *A. flavus* [8], respectively. Unique regions represent 1.2% and 2.3% (and harbour 143 and 218 genes) in the Af293 and A1163 genomes, respectively. More than half of the Af293-specific genes are also absent in *A. fumigatus* isolates Af294 and Af71, according to the array-based comparative genome hybridization (aCGH) data [7]. The vast majority of Af293- and A1163-specific genes are clustered together in blocks ranging in size from 10 to 400 Kb, which seem to be the most variable segment of the species genome. A manual examination of these isolate-specific islands revealed that they contain numerous pseudogenes and repeat elements. One of the regions contains a putative secondary metabolism cluster (AFUA_3G02530-AFUA3G02670).

The origin of 20% of Af293-specific genes can be attributed to two segmental duplication events. One of the duplicated regions (AFUA_1G16010- AFUA_1G16170) contains an arsenic detoxification cluster. The other (AFUA_1G00420-AFUA_1G00580) contains genes that may be involved in metabolism of betaine, which is often synthesized under osmotic and heavy metal stress. Interestingly the duplicated regions are also absent in Af294 and Af71 isolates, which suggests that the duplication event took place very recently.

Segmental duplication events are thought to contribute to rapid adaptation of the species by increasing their expression. Since Af293 is a clinical isolate it is possible that these chromosomal aberrations were created due to selective pressures in the host.

### Highly Variable Loci in *A. fumigatus*

Although most Af293 proteins are 100% identical to their A1163 orthologs, we have identified 41 orthologous pairs that share only 37% to 95% identity. To find out if these genes are also divergent in other *A. fumigatus* isolates, we identified Af293 genes that do not hybridize with DNA extracted from the Af294 and Af71 strains in aCGH experiments [7]. The comparison revealed that 27 out of 41 genes were possibly polymorphic (marked as absent or divergent) with respect to at least one other isolate (Table S3). Further analysis of three polymorphic loci in other *A. fumigatus* isolates has demonstrated that each of them harbours two or three alleles (Table S4). A PCR survey followed by Southern blot analysis and partial DNA sequencing has shown the presence of at least two alleles at each locus containing nearly identical sequences within each group of alleles (data not shown).

In filamentous fungi, this high level of variability has been previously associated with heterokaryon incompatibility (*het*) genes involved in a programmed cell death (PCD) pathway triggered by hyphal fusion between two genetically incompatible individuals [24],[25]. So far several *het* loci have been described in *A. nidulans* [26], although none have been characterized at the molecular level. Incidentally, our results are consistent with previously identified vegetative incompatibility groups suggesting that some of these polymorphic genes may function in heterokaryon incompatibility in *A. fumigatus*. Thus, four clinical isolates from the same multi-member incompatibility group (WSA-270, WSA-1195, WSA-449, and WSA-172) contained the same alleles of the polymorphic genes (Table S4).

**Figure 4:**
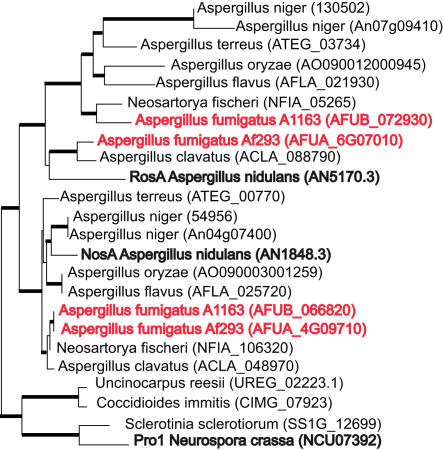
The Af293 RosA and NosA Proteins. Shown in bold red are RosA, NosA and Pro1 proteins that have been experimentally characterized are shown in bold black. Branches with a bootstrap of 75% or more are indicated in bold black. The trees are maximum-likelihood trees (see Materials and Methods).

Furthermore, at least five putative *A. fumigatus het* genes exhibit a pattern of trans-species (or trans-specific) polymorphism (Table S5), which has been previously associated with somatic and sexual incompatibility in fungi, self-incompatibility in plants, and the major histocompatibility complex (MHC) in vertebrates. These genes are more similar to their orthologs from other *Aspergillus* species than to those from A1163. We chose one putative *het* gene, *rosA* (AFUA_1G15910), and its close relative, *nosA* (AFUA_4G09710), whose orthologs encode two Zn2C6 transcriptional regulators of sexual development in *A. nidulans* [27],[28] for phylogenetic analysis (Figure 4). Unexpectedly, Af293 RosA clusters with its *A. clavatus* ortholog, while A1163 RosA clusters with *N. fischeri*. This is in contrast with the NosA tree, which perfectly mirrors the species tree (Figure 2), suggesting that these allelic classes may transcend species boundaries in the aspergilli.

This is the first study that shows the diversity of *het* genes in aspergilli at the molecular level as well as patterns of trans-species polymorphism. These putative *het* genes are distinct from those identified in *Neurospora crassa* or *Podospora anserina* [24],[25], although many of them share the same domains such as the NACHT and NB-ARC domains of the STAND superfamily [29]. Coincidentally four of the *A. fumigatus* variable genes encoding STAND domain proteins have previously been predicted to function in heterokaryon incompatibility [30]. The discovery of putative *het* loci in the aspergilli may facilitate identification of downstream components of fungal PCD pathways or other drug targets. These loci may be also used as a basis for classification of natural and clinical isolates into different compatibility groups.

### *A. fumigatus* vs. *N. fischeri* vs. *A. clavatus*

The genomes of *N. fischeri* and *A. clavatus* were sequenced by the whole genome sequencing method [22]. The *N. fischeri* genome (32.6 Mb) is 10–15% larger than the *A. clavatus* and *A. fumigatus* genomes (Table 1). There are 10,407 protein-coding genes and a large number of transposable elements, which may have contributed to its genome size expansion. The *A. clavatus* genome (27.9 Mb) is the smallest seen to date among the sequenced aspergilli (Table 1). There are currently 9,125 predicted protein-coding genes. This is consistent with past comparative studies that identified notable (up to 30%) genome size differences between distantly related aspergilli [7],[9],[10].

Despite this significant genome size variability, gene-level comparisons confirmed phylogenetic proximity of *A. fumigatus*, *N. fischeri* and *A. clavatus* (Figures 1 and 2). The three genomes also appear to be largely syntenic. Alignment of the *N. fischeri* and *A. clavatus* genomes against the eight Af293 chromosomes has revealed 20 and 55 syntenic blocks, respectively (Table 2). There is only one large-scale reciprocal translocation between chromosomes 2 and 5 in *N. fischeri* (blocks 8927.1, 8927.2, 9292.1 and 9292.2, in Figure 3). The *A. clavatus* supercontigs align with *A. fumigatus* chromosomes 2 and 5, suggesting that this was the ancestral topology.

**Table 2:** Syntenic and Afum-specific Chromosomal Blocks in Af293

Af293 blocks	Syntenic to A1163	Syntenic to *N. fischeri*	Syntenic to *A. clavatus*	Afum specific
No. of original blocks	29	24	62	13
No. of merged blocks	17	20	55	13
Merged blocks length	28.4 Mb	27.6 Mb	26.0 Mb	1.7 Mb
% Coding	50%	51%	52%	31%
Repeat^a^ density	0.51%	0.50%	0.47%	1.83%
TE^b^ density	1.07%	0.96%	0.80%	4.17%

Syntenic blocks for each pair of genomes were defined as areas containing a minimum of five orthologous genes in the Af293 and target genomes with a maximum of 20 adjacent non-matching genes. Afum-specific blocks were defined as Af293 areas containing at least ten Afum-specific genes and separated by no more than 5 other genes. Since most syntenic regions slightly overlap, the original blocks were merged to calculate repeat and TE density. Abbreviations: ^a^repeat elements; ^b^transposable elements. Repeat and TE densities were estimated as described in Materials and Methods.

### Core and Lineage-Specific Genes

#### Features of Core and Lineage-Specific Genes

**Figure 5:**
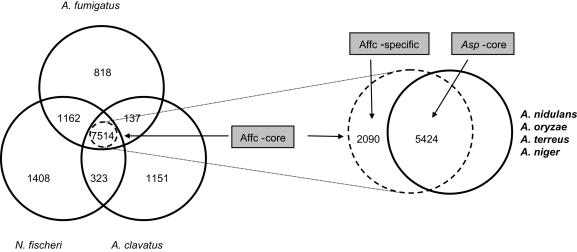
Proteins with Orthologs in the Three Most Closely Related Aspergilli (*A. fumigatus*, *N. fischeri* and *A. clavatus*). These proteins constitute the Affc-core group, and proteins with no orthologs in *N. fischeri* and *A. clavatus* constitute the *A. fumigatus*-specific group (Afum). The proteins in the Affc-core can be further divided into two groups, Aspergillus-core (Asp-core), which has orthologs in all of the other aspergilli, and the Affc-specific group, which is comprised of the rest of the Affc-core.

Comparative genomic analysis has showed that the three *Aspergillus* genomes contain a large number of species-specific genes, which is consistent with previous comparative studies [7]. We have identified 7514 orthologous core and 818, 1402 and 1151 species-specific genes in the Af293, *N. fischeri* and *A. clavatus* genomes, respectively (Figure 5). Numbers of core- and species-specific genes, however, depend on selection of genomes from which they were derived. Thus, adding new genomes to this comparison resulted in fewer core and specific genes as shown for Af293 in Table S6. The availability of additional sequenced *Aspergillus* genomes allowed us to explore these patterns in a more systematic manner by comparing *A. fumigatus* Af293 genes with different lineage specificity (i.e. number of orthologs in other species).

To this end, we have selected four sets of genes based on the presence of orthologs in the six other sequenced aspergilli: *N. fischeri*, *A. clavatus*, *A. terreus* (CH476594), *A. oryzae* [10], *A. nidulans* [9] and *A. niger* CBS 513.88 [23] (Table S6; Figure 5). Genes with orthologs in the three most closely related aspergilli (*A. fumigatus*, *N. fischeri* and *A. clavatus*) constitute the Affc-core group. The genes in the Affc-core can be further divided into two groups, the *Aspergillus*-core (Asp-core) with orthologs in all six other aspergilli and the Affc-specific group, which is comprised of the remaining Affc-core genes. Finally, the *A. fumigatus*-specific (Afum-specific) group contains Af293 genes that have orthologs in neither *N. fischeri* nor *A. clavatus*.

One of the most striking observations to arise from this comparison was the marked differences in size and number of exons among genes from different lineage-specificity groups (Table 3). For example, Asp-core genes on average are almost twice as large as Afum-specific genes. The latter have on average only 1.35 introns and almost 31% lack introns completely. In contrast, Asp-core genes contain on average 2.16 introns, only 16% of them without introns. Consistent with previous reports of increased evolutionary rates in LS genes (e.g. [31]), Affc- and Afum-specific genes in *A. fumigatus* exhibit low sequence identity to their orthologs from more distantly related fungi (Table 3).

**Table 3:** Comparison of Four Af293 Gene Sets with Different Lineage Specificity

Lineage specificity group	Asp-core	Affc-core	Affc-specific	Afum-specific
No of genes	5424	7514	2090	818
No of orthologs in 6 aspergilli	6	2–6	2–5	0–1
Mean gene length	1722	1579	1209	802
Mean No. of introns	2.16	2.02	1.66	1.35
%Genes without introns	15.9%	19.4%	28.5%	31.4%
% Affc syntenic	98.3%	96.0%	89.8%	n/a
% Telomere-proximal	5.6%	9.1%	38.0%	36.5%
% Expressed	42.5%	42.7%	43.3%	32.4%
% Orthologs in *A. clavatus*	100%	100%	100%	n/a
% Orthologs in *N. crassa*	81.5%	70.7%	42.6%	4.5%
% Orthologs in *S. cerevisiae*	49.9%	41.5%	19.9%	1.2%
% Identity to *A. clavatus* orthologs	81.3%	78.6%	71.4%	n/a
% Identity to *N. crassa* orthologs	52.3%	51.6%	47.9%	43.3%
% Identity to *S. cerevisiae* orthologs	43.1%	42.7%	40.4%	38.0%

The numbers of Af293 genes in different categories are shown for *Aspergillus*-core (Asp-core), Affc-core, Affc-specific, and *A. fumigatus*-specific (Afum-specific) groups (see main text for definitions). Telomere-proximal genes are defined as genes located within 300 Kb from the chromosome end. Affc syntenic genes are defined as Af293 genes syntenic with respect to *N. fischeri* and *A. clavatus* (see the legend to Table 2). The ‘expressed’ genes are defined as Af293 genes that showed differential expression in at least one microarray study (W. Nierman, unpublished).

**Figure 6:**
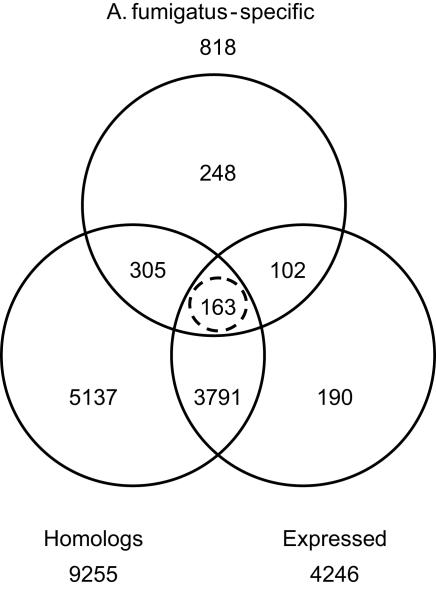
*A. fumigatus*-Species Specific Genes Supported by Homology and Expression Data. Genes with no orthologs in *N. fischeri* and *A. clavatus* constitute the *A. fumigatus*-specific group (Afum). Genes that have homologs in other fungal genomes constitute the Homology group. Genes differentially expressed in microarray studies represent the Expressed group.

These vast differences in gene features between core and specific genes are more likely to be explained by relaxed selective constraints (as discussed below) than by poor annotation quality of LS genes (due to misannotated gene models, gene fragments or random ORFs). We made significant improvements to Af294 gene models by leveraging the comparative genomic data (see Materials and Methods). In addition, all Affc-specific genes have orthologs in *N. fischeri* and *A. clavatus* and 43% of them are differentially expressed in various expression studies, which is similar to the *A. fumigatus* genome average (Table 3). On the other hand, many Afum-specific genes may be non-functional, since only 32% of them are differentially expressed in microarray studies (vs. the 43% genome average) and only 60% of them show sequence similarity to other fungal proteins (Table S7; Figure 6). Nonetheless, at least 20% of Afum-specific genes are supported by combined evidence (homology and expression data) and therefore are likely to be functional. Nonetheless, even these genes are still smaller in size than average Affc- and Asp-core genes.

#### Biological Roles and Chromosomal Location of LS Genes

Analysis of Gene Ontology (GO) terms [32] associated with core and lineage-specific groups has demonstrated that certain biological functions are unequally distributed among these groups (Table S8). The Afum-specific group is enriched for genes involved in carbohydrate transport and catabolism, secondary metabolite biosynthesis, and detoxification. In contrast, the invariable Asp-core genome encodes many functions associated with information processing and other cellular processes that contribute to the organism's fitness in most environments. Thus, a significant number of Asp-core genes (15%) are orthologous to yeast essential genes, which represents a two-fold enrichment in comparison to the rest of the proteome.

Although most Af293 genes involved in carbohydrate transport and catabolism are found in the Asp-core group, only 10% of secondary metabolism genes have orthologs in all sequenced aspergilli including siderophore, pigment and Pes1-related clusters. These three conserved clusters are also found in *Penicillium* species and some more distantly related fungi. Similarly, only 30% of secondary metabolism Af293 genes are shared by *N. fischeri* and *A. clavatus*. The three species also vary considerably in the numbers of enzymes that control the first step in secondary metabolite biosynthesis such as nonribosomal peptide synthases (NRPS), polyketide synthases (PKS), and dimethylallyltryptophan synthases (DMATS) (Table S9). Interestingly, *N. fischeri* genome contains 46 enzymes, which is 35% more than *A. clavatus* (35) and *A. fumigatus* (34) genomes.

Likewise, PFAM domains overrepresented among Affc- and Afum-specific genes have been shown to function in efflux or detoxification, secondary metabolite biosynthesis, resistance to antifungals, and other accessory metabolic pathways. They include MSF and ABC transporters, various oxidoreductases, cytochrome P450, glycosyl and alpha/beta fold hydrolases, polyketide synthases, glutathione transferases and methyltransferases (Table S10). On the other hand, core genes often contain AAA-superfamily ATPase, helicase, WD40, and SH3 domains associated with such important functions as cell organization and macromolecule biosynthesis.

#### Lineage Specific Genomic Islands

In addition to difference in size and function, lineage specific genes display a significant subtelomeric bias. As opposed to telomere-distal Asp- and Affc-core genes, Affc- and Afum-specific genes tend to be located within 300 Kb from chromosome ends (P value>0.01) (Table S11). About 38% of Affc-specific genes are telomere-proximal in comparison to 6% of Asp-core and 9% Affc-core genes (Table 3). Interestingly, 46% of Afum-specific genes with paralogs are telomere-proximal (Table S7), suggesting that they may have been recently duplicated and translocated to these regions. Our findings concur with previous reports of subtelomeric bias in LS genes in *A. fumigatus* [7], *S. cerevisiae* [33] and *Pichia stipitis* [34]. With the exception of one Af293 locus containing four P450 genes, the *Aspergillus* species do not have large variable subtelomeric arrays arising by a series of tandem duplications found in some protozoan parasites [35].

Almost 50% of the Afum-specific genes can be clustered together in 13 blocks containing more than 10 Afum-specific genes separated by no more than 5 genes outside this category (Table 2). Together these regions, referred to here as Afum-specific genomic islands, show an even more significant telomeric bias (68% of the clustered genes lay within 300 Kb from telomere ends) with larger blocks found almost exclusively at chromosome ends (Figure 3). In addition to non-syntenic genes, species-specific islands harbour a disproportionate number of transposons and other repeat elements in comparison with the syntenic areas of the Af293 genome (Table 2). Notably two *A. fumigatus*-specific blocks (2.2 and 3.1) contain gene clusters involved in biosynthesis of mycotoxin fumigaclavine and another unknown secondary metabolite [36]. Similar genomic islands have been described in the rice blast fungus *Magnaporthe oryzae* [37],[38] and in *A. oryzae* [10] suggesting that they may be shared across all filamentous ascomycota fungi. Unlike variable subtelomeric regions found in other eukaryotes [39],[40], these areas are often quite large (up to 400 Kb) and not always located near chromosome ends.

#### Evolutionary Origins of Lineage-Specific Genes

Most Affc- and Afum-specific genes have no orthologs in non-*Aspergillus* fungal species, which suggests that they were created *de novo* in the Affc lineage. To gain insight into the origin of the LS genes in aspergilli, we have performed phylogenetic analysis of two sets of *A. fumigatus-* and *N. fischeri-specific* genes. In Af293 and *N. fischeri*, Set 1 contains 790 and 1230 genes, respectively, that have an *Aspergillus* homolog as the best BLASTp hit; Set 2 contains 28 and 178 genes, respectively, that have a non-*Aspergillus* homolog as the closest relative. There is a significant difference in the numbers of trees including a non-*Aspergillus* species as the closest relative in *N. fischeri* and *A. fumigatus* (P value = 2.6e-08). This is indicative of major differences in retention and/or uptake of new genetic material in these two species, consistent with differences in their reproductive modes.

**Figure 7:**
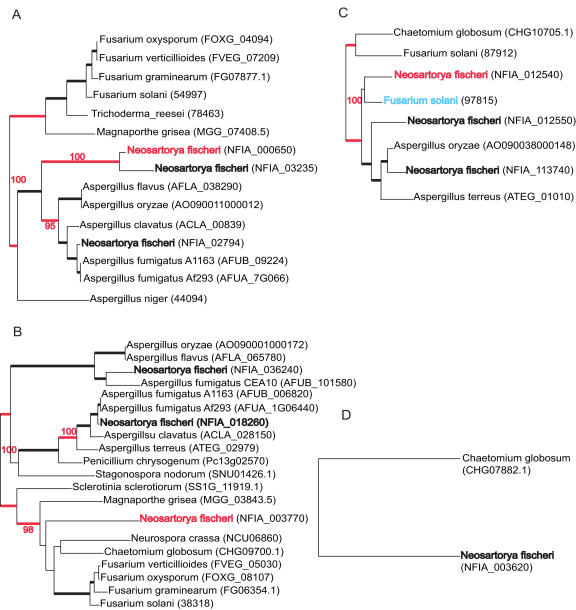
Four Common Topologies Detected by Phylogenetic Analysis of *N. fischeri*-Specific Proteins. The *N. fischeri* proteins under consideration are in bold red. The bootstrap supporting the clade containing the *N. fischeri* is also in bold red. Other *N. fischeri* proteins are shown in bold black. Blue species names correspond to the recipient genome when different from *N. fischeri*. Systematic gene names are indicated. Branches with a bootstrap of 75% or more are indicated in bold black. The trees are maximum-likelihood trees (see Materials and Methods). A. Set1 protein evolved by probable duplication, differentiation and differential loss in other Aspergillus species (DDL). B. Set 2 protein evolved by probable HGT from Sordaryomycetes into the *N. fischeri* lineage. C. Set 2 protein evolved by probable DDL and a *Fusarium solani* protein (in blue) evolved by probable HGT from the *N. fischeri* lineage into Sordaryomycetes. D. Set 2 protein showing similarity to a protein from the Sordaryomyce Chaetomium globosum.

The four repetitive scenarios identified by phylogenetic analysis are displayed in Figure 7. In both *A. fumigatus* and *N. fischeri*, most of the Set 1 genes exhibit topologies that do not strictly follow the *Aspergillus* species tree (Figure 2), although nested within the *Aspergillus* clade. Similarly, all 28 *A. fumigatus* Set 2 genes are nested within the *Aspergillus* genus. In contrast to the *A. fumigatus* genes, *N. fischeri* Set 2 genes sometimes cluster with a non-*Aspergillus* species with high bootstrap support. As shown in Figure 7B and 7C, both *N. fischeri* and non-*Aspergillus* species genes can be nested either in this non-*Aspergillus* clade or in the *Aspergillus* clade. At first sight, these repetitive topologies can be interpreted as supportive of a horizontal gene transfer (HGT) from a non-*Aspergillus* species into *N. fischeri* or visa versa. Further analysis, however, reveals that most of the conflicts involve sparsely populated trees, long branch attraction artifacts, and other situations, where phylogenetic methods tend to mislead (e.g. [41]). The last repetitive scenario includes genes that are only present in one other distant fungal genome (Figure 7D). The evolutionary origin of genes in this category cannot be resolved at this time.

Our results are consistent with the well established role of gene duplication and divergence as the principal source of new genes [42]–[45]. They are however in conflict with previous studies that attributed the origin of LS genes in the aspergilli to gene acquisition through HGT from other fungal species [9],[10],[46]. This assumption was based on circumstantial evidence such as mosaic phyletic distribution, phylogenetic anomalies, and differences in gene content among *A. fumigatus*, *A. nidulans* and *A. oryzae*. Besides the absence of readily apparent HGT examples, the fact that LS genes tend to be smaller in size and have fewer exons is difficult to explain by HGT. These gene features are quite consistent across *Aspergillus* species, and it is therefore unclear what could be the donor organism for LS genes.

The DDL scenario does not have this weakness, since these size differences can be a direct consequence of relaxed selective constraints operating on duplicate genes. According to the DDL hypothesis, the initial redundancy in gene function allows duplicate genes to quickly accumulate nonsynonymous mutations and even premature stop codons. Notably, over 20% of all Afum-specific genes can be linked to the two very recent segmental duplications events that occurred in Af293 but not in A1163. Both translocated segments are telomere-distal and contain genes that appear to be pseudogenized indicating that translocated gene copies may have evolved under relaxed selective constraints. Similarly in other species, accelerated evolution has been often associated with subtelomeric areas suggesting that the process is dependent on the local chromatin environment (e.g. [47]).

The prevailing role of duplication in the origin of LS genes in the aspergilli is further underlined by their tendency to cluster in genomic islands. These regions may function as designated “gene dumps” and simultaneously as “gene factories”, since some LS genes appear to maintain their functional integrity or at least are differentially expressed in microarray studies as shown above. As shown above, 46% of Afum-specific genes with paralogs are telomere-proximal (Table S7), suggesting that they may have been recently duplicated and translocated to these regions. Evidence for gene duplication and/or transfer to evolutionarily labile regions is found in some protozoan parasites that have large variable subtelomeric arrays arising by a series of tandem duplications [35].

### Conservation of Virulence-, Allergy-, and Sex-Associated Genes

Previous studies however have shown a high level of evolutionary conservation and phyletic retention among known *A. fumigatus* virulence-associated genes [7]. Our analysis confirmed the low rate of protein evolution among these genes in four *Aspergillus* species (Table S12). Interestingly, four of the virulence-associated genes, *pabaA* (AFUA_6G04820), *fos*-1 (AFUA_6G10240), *pes*1 (AFUA_1G10380) and *pksP* (AFUA_2G17600), reveal evidence of accelerated evolution in the branch leading to the two *A. fumigatus* isolates. This pattern can affect only a few amino acid residues (e.g. PksP) or a significant proportion of the protein (e.g. Pes1).

Such a pattern can be due to either relaxation of selection or selection for rapid diversification (positive selection). In the latter case specific amino acid substitutions may decrease susceptibility to specific environmental challenges and thus enhance *A. fumigatus* virulence. These four genes are involved in oxidative stress or nutrient availability, which is consistent with the positive selection scenario. Indeed, PabaA is involved in biosynthesis of folate, an essential co-factor for DNA synthesis. Since PABA is apparently limited in the mammalian lung, a functional *pabaA* gene is required for virulence [48]. Fos1, a putative two-component histidine kinase, may play a role in the regulation of cell-wall assembly [49]. Finally, PksP and Pes1 are enzymes, which catalyze the first steps in biosynthesis of the spore pigment and an unknown non-ribosomal peptide, have been shown to mediate resistance to oxidative stress in addition to their role in *A. fumigatus* virulence [50],[51]. The inclusion of additional taxa in the analyses might clarify the significance of the observed differences.

This overall lack of variability among known virulence-associated factors suggests that yet unknown *A. fumigatus*-specific genes may contribute to its ability to survive in the human host. A recent microarray study demonstrated that the Affc-specific genes are over-represented among genes that are up-expressed in the neutropenic murine lung (Elaine Bignell submitted for publication). Many of them are found in chromosomal gene clusters associated with macromolecule catabolism and secondary metabolite biosynthesis. Similarly, clustered lineage-specific genes simultaneously induced in infected tissue have been observed in the ubiquitous maize pathogen *Ustilago maydis* [52] and some other species (for a recent review see [53]). Alternatively *A. fumigatus* virulence may be a combinatorial process, dependent on a pool of genes, which interact in various combinations in different genetic backgrounds as suggested previously [7]. Similar ‘ready-made’ virulence features have been described in other environmental pathogens such as *Pseudomonas aeruginosa* [54] and *Cryptococcus neoformans* [55],[56].

In addition to virulence factors, the *A. fumigatus* genome encodes 20 allergens (Table S13) and 25 proteins displaying significant sequence similarity to known fungal allergens (Table S14), some of which appear to contribute to its pathogenicity [57]. For example, *A. fumigatus* Asp f6 (AFUA_1G14550), also known as Mn^2+^-dependent superoxide dismutase (MnSOD), is specifically recognized by IgE from patients with allergic bronchopulmonary aspergillosis (ABPA) and is differentially expressed during germination [58]. The broad distribution of allergens among fungal taxa (Text S1) suggests that *A. fumigatus* possesses the same allergen complement as most other aspergilli and that its effect on hypersensitive individuals can be explained mostly by its ubiquity in the environment.

Our analysis has demonstrated that, similar to known virulence-associated genes, most sexual development genes appear to be under negative (purifying) selection in both sexual and asexual *Aspergillus* species (Text S1 and Table S15). More detailed analysis has revealed four genes in the *N. fisheri* lineage that may be under positive selection. This suggests that a few amino acid changes may enable sexuality in *N. fischeri*. The conservation of sex genes in asexual species is due to a latent sexuality, a recent loss of sexuality, pleiotropy, or parasexual recombination following heterokaryon formation as suggested previously [59],[60].

### Conclusions

Lineage-specific (LS) genes (i.e. genes with limited phylogenetic distribution of orthologs in related species) have been the focal point of many comparative genomic studies, because of the assumption that they may be responsible for phenotypic differences among species and niche adaptation. Our analyses of the genomes of *A. fumigatus* and the two closely related species, *N. fischeri and A. clavatus*, demonstrates that *A. fumigatus* may possess genetic determinants that allow it to establish a successful *in vivo* infection. LS genes that have no orthologs in the other two species comprise 8,5% of the *A. fumigatus* genome and often have accessory functions such as carbohydrate and amino acid metabolism, transport, detoxification, or secondary metabolite biosynthesis. Further analysis showed that these genes have distinct features (e.g. the small gene length and number of introns) and tend to cluster in subtelomeric genomic islands, which may function as “gene dumps/factories”. The phylogenies of LS genes, their subtelomeric bias and size differences are consistent with the DDL hypothesis stating that duplication being the primary genetic mechanism responsible for the origin of species-specific genes. The presence of genomic islands indicates that *A. fumigatus* and may possess sophisticated genetic mechanisms that facilitate its adaptation to heterogeneous environments such as soil or a living host.

## Materials and Methods

### Fungal Isolates

*A. fumigatus* Af293 (FGSC A1100) was isolated from patients with invasive aspergillosis [61]. *A. fumigatus* A1163 (FGSC A1163) is a derivative of *A. fumigatus* CEA17 converted to *pyrG*+ via the ectopic insertion of the *A. niger pyrG* gene [62],[63]. CEA17 is a uracil auxotroph of *A. fumigatus* clinical isolate CEA10 (CBS144.89). The type strains of *A. clavatus* (NRRL 1) and *N. fischeri* (NRRL 181) were used for sequencing and phenotypic characterization.

### Accession Numbers

The genome sequences of *A. clavatus*, *N. fischeri* and *A. fumigatus* A1163 were deposited to the GenBank under the following accession numbers: AAKD00000000, AAKE00000000 and ABDB00000000, respectively.

### Whole Genome Sequencing

A1163, *A. clavatus* and *N. fischeri* were sequenced using the whole genome shotgun method as previously described [22]. Random shotgun libraries of 2–3 Kb, 8–12 Kb and 50 Kb were constructed from genomic DNA from each strain, and DNA template was prepared for high-throughput sequencing using Big Dye Terminator chemistry (Applied Biosystems). Sequence data was assembled using Celera Assembler. For *A. fumigatus* A1163, scaffolds were compared to those of the first sequenced isolate, Af293 [7].

### Sequence Identity at the Nucleotide Level

A1163 assemblies larger than 5 Kb were aligned to the Af293 chromosomes using the MUMmer package (http://mummer.sourceforge.net/) [64]. Alignments longer than 100 Kb were used to determine average sequence identity to avoid highly repetitive and duplicated regions. The same approach was used to estimate sequence identity between *A. flavus* and *A. oryzae* and between the two sequenced *A. niger* strains.

### Gene Structure Annotation

The JCVI eukaryotic annotation pipeline was applied to the A1163, *A. clavatus* and *N. fischeri* assemblies (supercontigs) larger than 2 Kb as described earlier [7]. We used PASA [65] and EvidenceModeler [66] to generate consensus gene models based on predictions from several types of genefinders including GlimmerHMM, Genezilla, SNAP, Genewise and Twinscan. Putative pseudogenes, small species-specific genes (less than 50 amino acids), and gene models overlapping with transposable elements (TE) shown in Table S16 were excluded from the final gene lists.

### Repetitive Elements

Identification of repeat elements was performed using RepeatMasker (http://www.repeatmasker.org/), RepeatScout (http://repeatscout.bioprojects.org/), and Tandem Repeats Finder (http://tandem.bu.edu/trf/trf.html). Putative TEs (Table S16) were identified by Transposon-PSI (http://transposonpsi.sourceforge.net), a program that performs tBLASTn searches using a set of position specific scoring matrices (PSSMs) specific for different TE families. TE and repeat densities were calculated as the percentage of nucleotide bases in the regions of interest (i.e., syntenic or non-syntenic blocks) that overlap with a feature of the appropriate type (repeat or TE).

### *A. fumigatus* Annotation Improvements

We leveraged the comparative genomic data to significantly improve annotation quality of the Af293 genome, which was previously annotated with relatively little supporting evidence [7]. The refinement of initial annotation was performed using the Sybil software package (http://sybil.sourceforge.net/), which allows for rapid identification of discrepancies in gene structure among orthologs. The comparison with orthologous *N. fischeri* and *A. clavatus* genes resulted in significant changes to the Af293 gene catalogue. Over 1100 gene models were updated and 130 new genes were identified. Initial *A. fumigatus* A1163 gene models were also improved using the PASA pipeline, initially developed to align expressed sequence tag (EST) data onto genomic sequences [65]. The pipeline was adapted to automatically update A1163 gene models by aligning them against Af293 coding sequences (CDSs).

### Functional Annotation

We have performed transitive functional annotation from Af293 proteins to their A1163, *N. fischeri* and *A. clavatus* orthologs. Previously GO terms [32] were assigned to Af293 proteins based on sequence similarity to PFAM domains or experimentally characterized *S. cerevisiae* proteins [7]. Secondary metabolism gene clusters were identified using Secondary Metabolism Region Finder (SMURF) available at http://www.jcvi.org/smurf (Nora Khaldi, unpublished). The complete list of gene clusters can be downloaded at ftp://ftp.jcvi.org/pub/software/smurf/. Gene Ontology (GO) terms [32] were assigned as described in [7]

### Ortholog Identification

After extensive computational and manual refinement, the improved protein datasets were used to generate the final set of orthologs. Orthologous groups in *Aspergillus* genomes were identified using a reciprocal-best-BLAST-hit (RBH) approach with a cut-off of 1e-05. In addition to the A1163, *A. clavatus* and *N. fischeri* genomes, the previously sequenced genomes of Af293 [7], *A. terreus* NIH2624 (http://www.broad.mit.edu), *A. oryzae* RIB40 [10], *A. nidulans* FGSC A4 [9] and *A. niger* CBS 513.55 [23] were included in the comparative analysis. The results of this analysis, as well as synteny visualisation and comparative analysis tools can be also found in the *Aspergillus* Comparative database at http://www.tigr.org/sybil/asp. Orthologous, unique and divergent genes in Af293 were identified based on alignments of Af293 CDSs against A1163 assemblies using gmap as implemented in PASA [65] using default parameters.

### Synteny Analysis

Syntenic blocks for each pair of genomes (Af293 vs. *A. clavatus* and Af293 vs. *N. fischeri*) were defined as areas containing a minimum of five matching (orthologous) genes with a maximum of 20 adjacent non-matching genes (having no orthologs) in the reference and target genomes. Since most syntenic regions slightly overlapped, the original blocks were merged to calculate repeat and TE density. Af293 non-syntenic blocks were defined as areas excluded from the syntenic blocks and containing at least ten Af239 non-matching genes.

### Statistical Analysis

Genes in four lineage-specificity groups were analyzed by the EASE module [67] in MEV within TM4 (http://TM4.org) [68] to identify overrepresented Gene Ontology (GO) terms, Pfam domains and Chromosomal Regions (telomere-proximal and central). Only categories with Fisher's exact test probabilities above with P>0.05 from the EASE analyses were reported for each gene set.

### Selective Constraints

Selective constraints were estimated for sets of orthologous genes from the Af293, A1163, *A. clavatus*, *N. fischeri* and *A. terreus* genomes. The rate of substitution in synonymous (*d*_S_) and in non-synonymous (*d*_N_) sites, and their ratio (*d*_N_/*d*_S_) was calculated using the PAML package [69]. If a gene is very well conserved, *d*_N_/*d*_S_<0.1; if a gene is under weak purifying selection, 0.1<*d*_N_/*d*_S_<1; if a gene is evolving neutrally (e.g. pseudogenes), *d*_N_/*d*_S_∼ = 1; and if a gene is evolving under diversifying selection, *d*_N_/*d*_S_>1. The results are reported only for orthologous genes sets having unsaturated *d*_S_ values, the same number of exons, and sequence alignment coverage >95%. For each gene, the average *d*_N_/*d*_S_ ratio for five pairwise species comparisons was calculated.

### Phylogenetic Analyses

We assembled a local database of protein sequences from the 28 publicly available fungal genome projects (Table S17). All phylogenetic analyses in this paper were carried out on protein sequences. The *A. niger* ATCC 1015, *Nectria haematococca*, *Phanerochaete chrysosporium* and *Trichoderma reesei* genomes projects was completed under the auspices of the US Department of Energy's Office of Science, Biological and Environmental Research Program and the by the University of California, Lawrence Livermore National Laboratory (Contract No. W-7405-Eng-48), Lawrence Berkeley National Laboratory (contract No. DE-AC03-76SF00098) and Los Alamos National Laboratory (contract No. W-7405-ENG-36).

To produce a reference tree of species phylogeny we used the protein sequences of 90 likely orthologs from *A. niger*, *A. nidulans*, *A. terreus*, *A. oryzae*, *A. clavatus*, *N. fischeri*, *A. fumigatus* and *Fusarium graminearum* (teleomorph of *Gibberella zeae*) as an outgroup. To minimize the effect of incorrect or incongruent gene models, these proteins were chosen on the basis of having identical numbers of introns in each species and similar lengths. Sequences were aligned using MUSCLE [70] and columns of low conservation were removed manually. Maximum-likelihood trees were constructed using the PHYLIP package, applying the JTT substitution model with a gamma distribution (alpha = 0.5) of rates over four categories of variable sites.

Phylogenetic analyses of individual Af293, A1163, and *N. fischeri* proteins were carried out on sets of homologs identified in BLASTP searches against our fungal database. The top 20 hits with E<10^−4^ were retained for analysis. Sequences were aligned using ClustalW [71]. Poorly aligned regions were removed using Gblocks [72]. Finally, a maximum likelihood tree was drawn using PHYML [73].

### Southern Blot Analysis

To detect polymorphisms in the *rosA* (AFUA_6G07010) gene, several hybridizations were performed using *rosA* gene as the probe and genomic DNA cleaved with *EcoR*I, *ClaI*, *BamH*I or *EcoR*V. For comparison, an invariable gene for all species (*apg5*; AFUA_6G07040) was used as the hybridization probe on genomic DNA digested with *Hpa*I.

### Colony Radial Growth Rate Measurement

Colony radial growth rate measurements were performed as described [74]. For each isolate, four (90 mm diameter) Petri dishes containing 25 ml agar medium were inoculated centrally with 2.5 µl of 1×10^6^ spores/ml suspension in PBS/Tween 80. Plates were then incubated at temperatures ranging from 25°C to 50°C and colony edges were marked using a plate microscope. Colonies were marked twice daily for 4–5 days. For each colony, two diameters perpendicular to each other were measured.

Eight replicates were measured for each isolate. The results reported here are the mean of two experiments. At least five time points during the log phase were used to calculate growth rate. The radius of the colonies was plotted against time using least-square regression analysis, and the slope of the regression line, which represents the growth rate, was calculated. Each replicate was analysed separately and the mean of the growth rate was then calculated.

## Supporting Information

Text S1 Allergens and sexual development genes.
(0.05 MB DOC)

Table S1 Growth rates of Af293, A1163, *N. fischeri*, and *A. clavatus* isolates at various temperatures.
(0.02 MB XLS)

Table S2 Resistance to antifungals among *A. fumigatus* clinical isolates.
(0.02 MB XLS)

Table S3 Divergent *A. fumigatus* Af293 genes with respect to Af294, Af71, and A1163.
(0.03 MB XLS)

Table S4 Distribution of polymorphic alleles among *A. fumigatus* isolates.
(0.02 MB XLS)

Table S5 Five *A. fumigatus* loci exhibiting trans-species polymorphism.
(0.02 MB XLS)

Table S6 *A. fumigatus* core and species-specific genes.
(0.02 MB XLS)

Table S7 Features of *A. fumigatus*-specific genes.
(0.02 MB XLS)

Table S8 Top biological processes overrepresented among four lineage specificity groups.
(0.02 MB XLS)

Table S9 Enzymes that control the first step in secondary metabolite biosynthesis.
(0.02 MB XLS)

Table S10 Top PFAM domains overrepresented among four lineage specificity groups.
(0.02 MB XLS)

Table S11 Lineage specificity and chromosomal location.
(0.02 MB XLS)

Table S12 Selective constraints operating on virulence-associated genes.
(0.02 MB XLS)

Table S13 Known *A. fumigatus* Af293 allergens.
(0.02 MB XLS)

Table S14 Predicted *A. fumigatus* Af293 allergens.
(0.02 MB XLS)

Table S15 Selective constraints operating on sex genes.
(0.02 MB XLS)

Table S16 Families of transposable elements identified in the Affc genomes.
(0.01 MB XLS)

Table S17 Fungal genomes used in phylogenetic analyses.
(0.02 MB XLS)
