# Supplementary material for: Genomic Islands in the Pathogenic Filamentous Fungus Aspergillus fumigatus
Source: PLoS Genet. 2008 Apr 11;4(4):e1000046. doi: 10.1371/journal.pgen.1000046 (PMC2289846; doi:10.1371/journal.pgen.1000046)
Supplement: Text S1 — Allergens and sexual development genes. (0.05 MB DOC) [file pgen.1000046.s001.doc]

**Allergens**

Our analysis (Tables S13 and S14) confirmed that the core set of known and predicted allergens is conserved in all sequenced *Aspergillus* species. Yet despite the high level of overall sequence conservation, some allergens contain divergent regions in the center or at the C-terminal end of the predicted protein. These regions appear to be under positive selection, but the selective constraints were difficult to estimate due to poor alignment quality. The list includes putative cell wall (GPI or glucanase type) allergens, typified by Asp f 2 ([AFUA_4G09580](http://www.ncbi.nlm.nih.gov/sites/entrez?Db=gene&Cmd=Retrieve&dopt=full_report&list_uids=3509208&log$=databasead&dbfrom=protein)), Asp f 4 ([AFUA_2G03830](http://www.ncbi.nlm.nih.gov/sites/entrez?Db=gene&Cmd=Retrieve&dopt=full_report&list_uids=3506748&log$=databasead&dbfrom=protein)), Asp f 7 ([AFUA_4G06670](http://www.ncbi.nlm.nih.gov/sites/entrez?Db=gene&Cmd=Retrieve&dopt=full_report&list_uids=3509245&log$=databasead&dbfrom=protein)), Asp f 9 ([AFUA_1G16190](http://www.ncbi.nlm.nih.gov/sites/entrez?Db=gene&Cmd=Retrieve&dopt=full_report&list_uids=3510010&log$=databasead&dbfrom=protein)) and Asp f 17 ([AFUA_4G03240](http://www.ncbi.nlm.nih.gov/sites/entrez?Db=gene&Cmd=Retrieve&dopt=full_report&list_uids=3504039&log$=databasead&dbfrom=protein)). Some of these proteins are members of the adhesion family and therefore such differences in structure may reflect differences in adhesion substrate between the various aspergilli. Additionally the variable domains of these proteins may reduce the likelihood of cross-reactivity to IgE from non-*A. fumigatus* species.

**Sexual Development Genes**

Sexual reproduction has never been observed in *A. fumigatus*, which reproduces clonally generating haploid conidia from aerial conidiospores [1,2]. In addition, *A. fumigatus* has other characteristics of a sexual species such as the presence of isolates of opposite mating type in similar proportions in nature [3,4]. The organization of the *MAT* loci of Af293 and A1163 is consistent with a heterothallic breeding system, with the two sequenced strains being of complementary mating types containing HMG- ([AFUA_3G06170](http://www.ncbi.nlm.nih.gov/sites/entrez?Db=gene&Cmd=Retrieve&dopt=full_report&list_uids=3512023&log$=databasead&dbfrom=protein)) and alpha- (AFUB_042900) domain encoding *MAT* genes respectively. The *A. clavatus* genome has only one *MAT* locus, containing an alpha *MAT* gene ([ACLA_034110](http://www.ncbi.nlm.nih.gov/sites/entrez?Db=gene&Cmd=Retrieve&dopt=full_report&list_uids=4702799&log$=databasead&dbfrom=protein)), suggesting that it can also be a heterothallic species. In contrast, the sequenced strain (NRRL181) of homothallic fungus *N. fischeri* has two unlinked *MAT* loci as was determined previously for another strain, NRRL4075 [5].

Interestingly *N. fischeri* *MAT2* ([NFIA_024390](http://www.ncbi.nlm.nih.gov/sites/entrez?Db=gene&Cmd=Retrieve&dopt=full_report&list_uids=4590586&log$=databasead&dbfrom=protein)) is found in a *N. fischeri*-specific island and has probably arisen by a TE-assisted translocation of a chromosomal region in a heterothallic ancestral species. Similarly in another homothallic species, *A. nidulans*, two unlinked *MAT* loci were separated by an apparent chromosome translocation [6]. These observations suggest that transitions between hetero- and homothallism typically involve recombination at regions flanking the *MAT* loci sometimes followed by translocation.

In addition to the HMG mating-type gene, both *N. fischeri MAT2* and Af293 *MAT* loci were found to contain a small uncharacterized gene ([NFIA_024400](http://www.ncbi.nlm.nih.gov/sites/entrez?Db=gene&Cmd=Retrieve&dopt=full_report&list_uids=4590587&log$=databasead&dbfrom=protein) and [AFUA_3G06160](http://www.ncbi.nlm.nih.gov/sites/entrez?Db=gene&Cmd=Retrieve&dopt=full_report&list_uids=3512260&log$=databasead&dbfrom=protein), respectively). This putative gene occupies the same position, but shares no apparent sequence similarity with, the *N. fischeri* and *A. clavatus* alpha *MAT* genes ([NFIA_071100](http://www.ncbi.nlm.nih.gov/sites/entrez?Db=gene&Cmd=Retrieve&dopt=full_report&list_uids=4590482&log$=databasead&dbfrom=protein) and [ACLA_034110](http://www.ncbi.nlm.nih.gov/sites/entrez?Db=gene&Cmd=Retrieve&dopt=full_report&list_uids=4702799&log$=databasead&dbfrom=protein), respectively). This putative mating-type gene had only one identifiable homolog in the public databases, a hypothetical protein ([CIMG_00407](http://www.ncbi.nlm.nih.gov/sites/entrez?Db=gene&Cmd=Retrieve&dopt=full_report&list_uids=4566037&log$=databasead&dbfrom=protein)), which is found in the *MAT* locus of the heterothallic ascomycete fungus *Coccidioides immitis* [7].

Further comparison of the *A. fumigatus*, *N. fischeri* and *A. clavatus* genomes confirmed that, despite different reproductive modes, all possess a full complement of previously identified euascomycete sex genes. The divergence of these genes at the amino acid level is similar to the average for these genomes. To estimate selective constraints operating on these genes, we selected orthologous Af923, A1163, *N. fischeri*, *A. clavatus* and *A. terreus* genes with validated gene structures (see Materials and Methods). The analysis has shown that *d*_N_/*d*_S_ ratios are very low, which is indicative of strong negative selection (Table S15). The only exception involves the *rosA* gene ([AFUA_6G07010](http://www.ncbi.nlm.nih.gov/sites/entrez?Db=gene&Cmd=Retrieve&dopt=full_report&list_uids=3508850&log$=databasead&dbfrom=protein)), which is located in a highly polymorphic locus and is probably under balancing selection (see Results and Discussion).

Four genes in the *N. fisheri* lineage seem to be under positive selection including three proteins involved in maturation and export of a-pheromone and G protein complex beta subunit SfaD ([AFUA_5G12210](http://www.ncbi.nlm.nih.gov/sites/entrez?Db=gene&Cmd=Retrieve&dopt=full_report&list_uids=3511647&log$=databasead&dbfrom=protein); see Table S15). This suggests that a few amino acid changes may enable sexuality in *N. fischeri*, and that the conservation of other regions in these genes in asexual species is due to pleiotropy. On the other hand, these genes may be involved in some other cellular function such as hyphal fusion during vegetative growth, ploidy reduction during interspecies mating, or asexual development. The presence of four genes exhibiting different selective constrains in *N. fisheri* might suggest the latter, i.e., that these are pleiotropic genes, and that a few selected amino acid changes might confer active sexuality. In any case, better understanding of sexual development and the evolution of sex in the aspergilli may facilitate experimental studies of *A. fumigatus* and related species by turning them into genetically tractable organisms with amenable sexual cycles.

**References**

1. Pringle A, Baker DM, Platt JL, Wares JP, Latge JP, et al. (2005) Cryptic speciation in the cosmopolitan and clonal human pathogenic fungus Aspergillus fumigatus. Evolution Int J Org Evolution 59: 1886-1899.

2. Rydholm C, Szakacs G, Lutzoni F (2006) Low genetic variation and no detectable population structure in aspergillus fumigatus compared to closely related Neosartorya species. Eukaryot Cell 5: 650-657.

3. Nierman WC, Pain A, Anderson MJ, Wortman JR, Kim HS, et al. (2005) Genomic sequence of the pathogenic and allergenic filamentous fungus Aspergillus fumigatus. Nature 438: 1151-1156.

4. Paoletti M, Rydholm C, Schwier EU, Anderson MJ, Szakacs G, et al. (2005) Evidence for sexuality in the opportunistic fungal pathogen Aspergillus fumigatus. Curr Biol 15: 1242-1248.

5. Rydholm C, Dyer PS, Lutzoni F (2007) DNA Sequence Characterization and Molecular Evolution of MAT1 and MAT2 Mating-Type Loci of the Self-Compatible Ascomycete Mold Neosartorya fischeri. Eukaryot Cell 6: 868-874.

6. Galagan JE, et al. (2005) Sequencing of Aspergillus nidulans and comparative analysis with A. fumigatus and A. oryzae. Nature 438: 1105-1115.

7. Mandel MA, Barker BM, Kroken S, Rounsley SD, Orbach MJ (2007) Genomic and population analyses of the mating type loci in Coccidioides species reveal evidence for sexual reproduction and gene acquisition. Eukaryot Cell 6: 1189-1199.
